# Longitudinal source-sink dynamics of fecal litter and farm indoor environmental resistomes in broiler chicken and Cherry Valley ducks

**DOI:** 10.1186/s42523-026-00544-x

**Published:** 2026-03-09

**Authors:** Peter Fauszt, Maja Mikolas, Peter David, Zsombor Szoke, Njomza Gashi, Emese Szilagyi-Tolnai, Endre Szilágyi, Maria Magdolna Szarvas, Monika Eva Fazekas, Andrea Kun-Nemes, Aniko Stagel, Ferenc Gal, Levente Czegledi, Sandor Biro, Laszlo Stundl, Judit Remenyik, Melinda Paholcsek

**Affiliations:** 1 Complex Systems and Microbiome-innovations Centre, Faculty of Agricultural and Food Sciences and Environmental Management, University of Debrecen, Debrecen, Hungary; 2 Hungarian National Blood Transfusion Service Nucleic Acid Testing Laboratory, Budapest, Hungary; 3 Department of Animal Husbandry, Faculty of Agricultural and Food Sciences and Environmental Management, Institute of Animal Science, Biotechnology and Nature Conservation, University of Debrecen, Debrecen, Hungary; 4 Department of Human Genetics, Faculty of Medicine, University of Debrecen, Debrecen, Hungary; 5 Faculty of Agricultural and Food Sciences and Environmental Management, University of Debrecen, Debrecen, Hungary

**Keywords:** Broiler, Duck, Microbiome, Antimicrobial resistance, Farm environment

## Abstract

**Background:**

Antimicrobial resistance is a major One Health threat, and intensive poultry systems function as amplifiers. Although broilers and ducks are reared under similarly controlled conditions, their microecologies diverge. Integrated, longitudinal source-sink analyses quantifying overlap and directional flux between host-associated and environmental resistomes remain scarce. A two-year (2022–2024), longitudinal, commercial-scale comparison was undertaken across 15 stocking cycles under harmonized husbandry in Ross 308 broiler and Cherry Valley duck. Parallel shotgun metagenomics profiled fecal litter and farm indoor environments across standardized production, with daily monitoring in one complete cycle per system; in total, 96 pooled samples were sequenced to quantify cross-compartment overlaps.

**Results:**

Antibiotic resistance gene (ARG) reservoir dominance proved to be system-specific, duck systems were environment-centric, whereas broiler systems were fecal litter-centric. Although overall ARG diversity was similar between systems (broiler 2,542; duck 2,494 types), ducks exhibited greater compartmental divergence, with ~ 2.6-fold more environment-unique ARGs than paired fecal litter and 1.15-fold higher environmental richness than broilers. Compartment coupling also differed: broilers showed tighter host-environment overlap, while ducks were more partitioned. A shared environmental ARG pool (57.5%) indicated substantial cross-system exchange potential. Temporally, shared ARGs accumulated across the grow-out and peaked pre-depopulation. The distribution of significant ARG carrier species revealed asymmetric host-environment coupling: overlap across compartments was 66.67% in broilers *versus* 45.45% in ducks, notably. The impact of antimicrobial use was nuanced: short, targeted courses were associated with lower aaAMR burden overall Collectively, the recurrent detection of clinically consequential carriers (*P. aeruginosa*, *E. coli*, *A. baumannii*, *S. aureus*, *K. pneumoniae*, *S. maltophilia*, toxigenic *Clostridium* spp.) underscored One Health risks of zoonotic spillover and food-chain contamination.

**Conclusion:**

Reservoir behavior in intensive poultry systems should be treated as system-specific, and matrix-targeted, with biofilm and humidity management prioritized in duck operations, and litter/manure control emphasized in broilers. The finisher-depopulation window emerges as a critical intervention point, warranting intensified mitigation clean-out. Finally, mitigation should extend beyond individual farms to transport crates, vehicles, shared equipment, and supply chains.

**Supplementary Information:**

The online version contains supplementary material available at 10.1186/s42523-026-00544-x.

## Background

Antimicrobial resistance is a defining public health threat of the 21st century, with consequences across human and veterinary medicine [1]. Broiler chickens and ducks, among the most widely farmed poultry in the EU and in Hungary, are central to this landscape [2].

Intensive production can amplify resistance emergence and spread, and flock-level antibiotic use, including prophylaxis, and continues to impose selection on farm microbiomes despite mitigation efforts [3–5]. The EU has tightened stewardship, and Hungary aligns through national antimicrobial use (AMU)/ antimicrobial resistance (AMR) surveillance and sector-specific strategies [3, 6–8].

Within farms, fecal litter-excreted resistant bacteria and antibiotic resistance genes (ARGs) constitute a major reservoir that seeds indoor environments via aerosolization and runoff, facilitating horizontal gene transfer (HGT) and sustaining transmission within and beyond production cycles [9–12]. Re-entry into the house ecology increases exposure for flocks, workers, and consumers, underscoring the need to understand source-sink dynamics: environmental compartments not only sequester fecal litter-derived ARGs but also act as secondary sources that drive recurrent re-inoculation and inter-compartment gene flow across animal-environment-human interfaces [3, 13].

Although both broiler chickens and Cherry Valley ducks are raised in closed, commercial-scale systems with standardized management and biosecurity, their microecologies diverge. Broiler houses run drier regimes with closed-nipple drinkers, concentrating pathogens and resistance in the fecal litter compartment [14, 15]. Duck houses, by contrast, sustain elevated moisture creating wet litter, splash zones, and waterline biofilms that stabilize aquatic opportunists, enhance horizontal gene transfer, and drive aerosol/fomite deposition [16, 17].

Thus, the farm indoor environment functions as a persistent reservoir in ducks, whereas broilers remain comparatively fecal litter-centric.

Also, the environment plays a dual role in the transmission dynamics of antimicrobial-resistant bacteria between poultry and humans. It functions both as a passive reservoir and as an active transmission medium, facilitating the dissemination of resistant pathogens through contaminated water, dust, and fomites [18, 19].

Antibiotic-resistant bacteria and their antibiotic ARGs can circulate through HGT between flocks and environmental reservoirs [20, 21]. Recent studies have increasingly emphasized the diverse mechanisms facilitating HGT, including conjugation, transformation, and transduction, which play critical roles in disseminating ARG genes within poultry production systems [22–24]. A central challenge in antimicrobial resistance research under intensive poultry production is the scarcity of integrative source-sink analyses that quantify overlap and directional flux between host-associated (fecal litter) and indoor environmental resistomes and the interactions between animal and environmental reservoirs, including cross-habitat gene flow and the conditions that sustain it, are still incompletely resolved.

Much of the literature interrogates a single compartment or a single species (often broilers), or narrows to target taxa (e.g., resistant *E. coli*) and isolated time points [3, 16, 25, 26].

Comparative studies exist (e.g., farm environment vs. human clinical isolates; backyard vs. intensive settings; metagenomic surveys across agricultural matrices; exposure studies linking poultry operations to human gut resistomes), yet side-by-side, longitudinal, dual-compartment assessments within the same production systems remain rare.

Our recent study closes this gap by implementing longitudinal, multi-compartment resistome profiling across 15 full production cycles in two rearing systems (broiler chicken and Cherry Valley duck), spanning antibiotic-treated and antibiotic-free cohorts.

Under harmonized husbandry, we aimed to perform parallel shotgun metagenomics on biological (fecal litter) and environmental matrices (indoor surfaces) to enable a system-level comparison that resolves ARG (i) reservoir dominance (fecal litter vs. indoor environment), (ii) host-environment coupling, (iii) diversity architecture, (iv) stage-resolved temporal dynamics (starter → grower → finisher), and (v) prophylactic antimicrobial-use effects.

## Results

### Description of the study

**Fig. 1:**
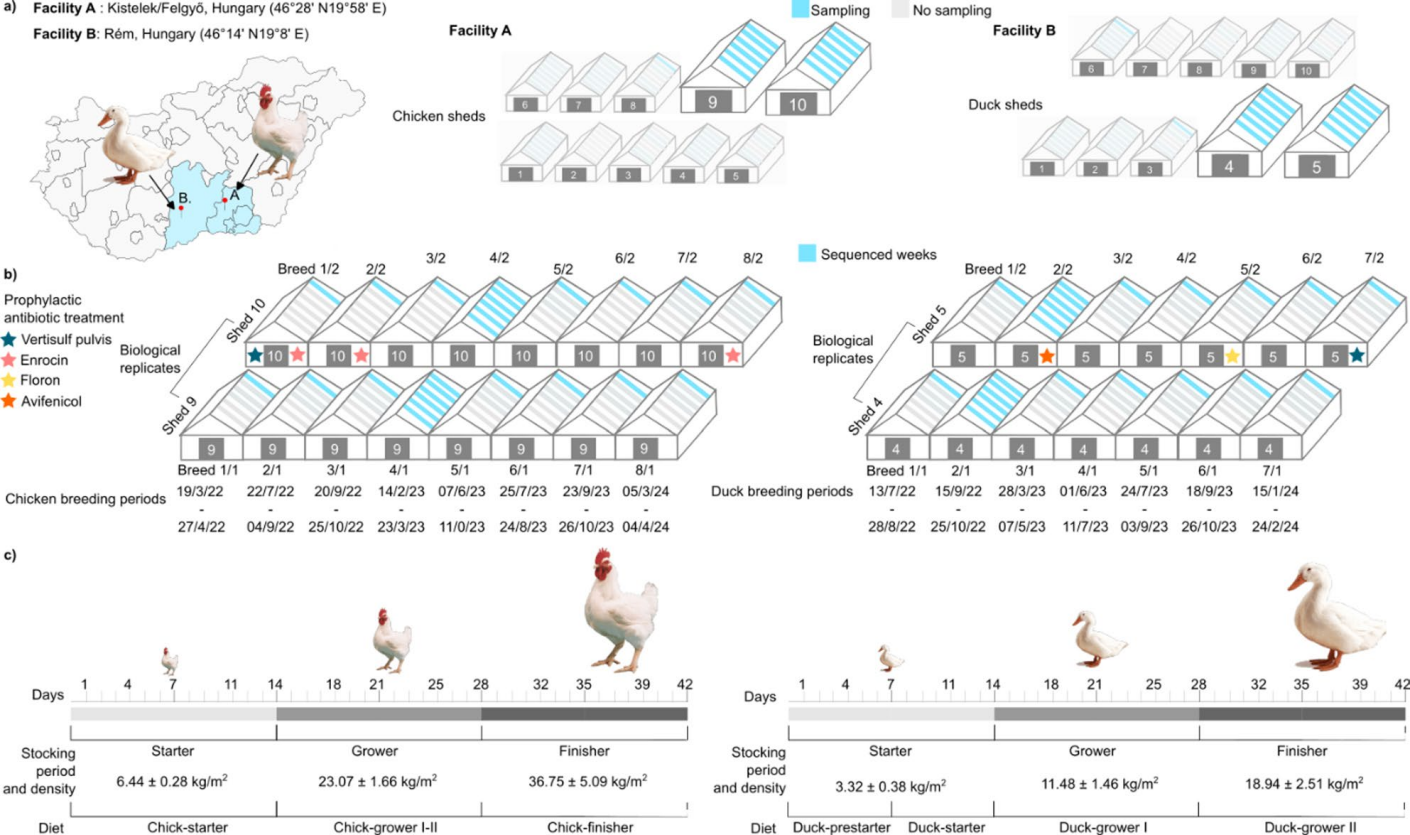
Overview of the experimental design, farm locations and sampling scheme for Ross 308 broiler chickens and Cherry Valley ducks raised under intensive production conditions in Hungary. (**a**) Broilers were reared in sheds 1–10 at two Hungarian sites, Kistelek and Felgyő (~ 30,000 birds/shed), and ducks in sheds 1–10 at Rém (~ 8,000 birds/shed). Sampled sheds are shaded blue (broiler 9–10; duck 4–5) and were randomly selected for sampling. (**b**) We monitored 15 production cycles in total (8 broiler, 7 duck). Sampling was daily with weekly pooling. One representative cycle per species, all weeks are marked in blue (broiler cycle 4; duck cycle 2) underwent full longitudinal sequencing of all six weekly pools (weeks 1–6); in the remaining cycles, only the week-6 (pre-slaughter) pools were sequenced (blue bars in the timelines). In total, 96 pooled samples were sequenced: 50 from broilers (26 fecal litter, 24 environmental) and 46 from ducks (24 fecal litter, 22 environmental). Prophylactic antibiotic courses occurred in three cycles per species and are indicated by colored stars (blue/red/yellow/orange). (**c**) Timeline of the production cycle, indicating the duration of each phase along with stocking density and diet information. Bird icons illustrate the growth of the birds throughout the cycle

The study focused on poultry raised under intensive farming conditions, specifically the two most commonly farmed meat-type avian species in Hungary: Ross 308 broiler chickens and Cherry Valley ducks. Broilers were reared at production sites in Kistelek and Felgyő, and ducks at a single site in Rém, all operated by Hungerit Zrt (Fig. 1).

Average flock sizes per cycle were 28,836 ± 2,806 for broilers and 7,137 ± 628 for ducks. Mortality rates remained low across all cycles, averaging 1,545 ± 490 for broilers and 513 ± 275 for ducks.

Stocking densities increased markedly over the course of each production cycle. In broilers, density rose from 6.44 kg/m² during the starter phase to 36.75 kg/m² in the finisher phase, an almost sixfold increase. For ducks, density increased from 3.32 kg/m² to 18.94 kg/m², representing an approximately sixfold rise.

Over the 15-month study period, a total of 15 production cycles were monitored per species (8 broiler and 7 duck cycles). In these cycles, daily sampling was performed throughout all six weeks, and all weekly pooled samples were subjected to sequencing. In the remaining cycles, daily sampling and weekly pooling were also conducted, but only samples from week 6, representing the pre-slaughter stage, were sequenced, allowing for standardized end-point comparisons.

In total, 96 pooled samples were collected: 50 from broiler cycles (26 fecal litter and 24 environmental) and 46 from duck cycles (24 fecal litter and 22 environmental). Notably, sequencing failed for two environmental samples (broiler cycle 4, Week 2 pools from 9th and 10th sheds, and duck cycle 2, Week 6 pools from 4th and 5th sheds) from each species. Consequently, the number of successfully sequenced environmental samples was reduced from the intended 26 to 24 in broilers and from 24 to 22 in ducks.

Prophylactic antibiotic treatments were administered in three broiler cycles (cycles 1, 2, and 8) and three duck cycles (cycles 2, 5, and 7), following veterinary prescription and EU regulatory guidelines. Ducks were treated with Vertisulf Pulvis and Enrocin (sulfonamides, diaminopyrimidines, fluoroquinolones), while broilers received Vertisulf Pulvis, Floron, and Avifenicol (sulfonamides, diaminopyrimidines, fluoroquinolones, phenicols). Treatments were applied at the maximum approved therapeutic doses and targeted specific rearing phases (e.g., starter or grower).

### Shotgun sequencing results

Shotgun metagenomic sequencing was performed using the Illumina NovaSeq platform (Illumina, USA). On average, we obtained 42,496,757 ± 7,338,415 reads per sample from ducks and 46,493,771 ± 8,826,263 reads from chickens. The average read counts by domain were as follows: *Archaea*: Duck: 1,062 ± 1,249; Chicken: 1,084 ± 3,444, *Bacteria*: Duck: 42,229,138 ± 7,418,026; Chicken: 45,799,522 ± 8,837,808, *Eukaryota*: Duck: 163,709 ± 406,924; Chicken: 492,977 ± 1,047,765, *Virus*: Duck: 102,848 ± 191,455; Chicken: 200,188 ± 327,869.

### Comparative analysis of the ARG load and richness in fecal litter and environmental samples

**Fig. 2:**
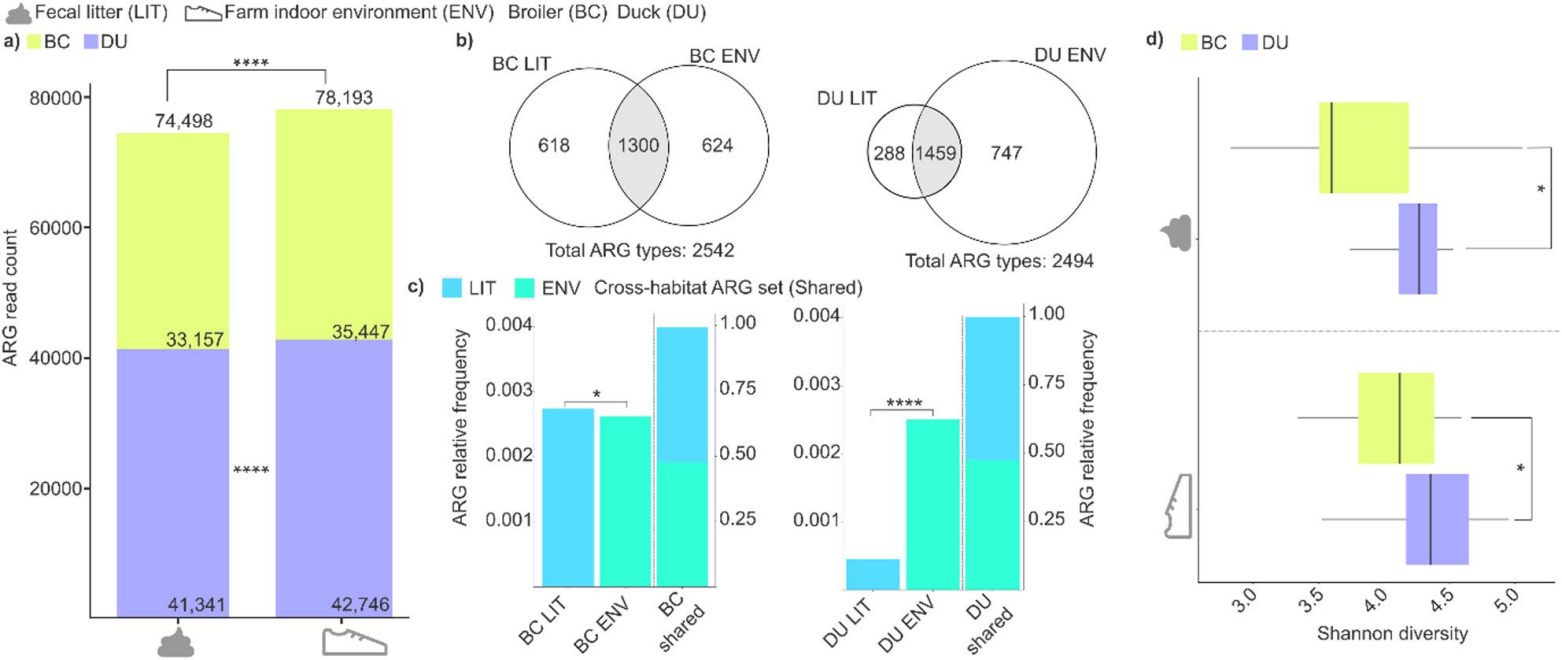
Resistome load, sharing patterns, and antibiotic resistance gene (ARG) richness across fecal litter (LIT) and farm indoor environment (ENV) in broiler (BC) and duck (DU) systems. (**a**) Stacked bars summarize cumulative ARG read counts and their distribution between LIT and ENV within each species; ****, *p* < 0.0001. (**b**) Proportional Venn diagrams show the numbers of unique and shared ARGs (non-redundant) between LIT and ENV for broilers and ducks; totals are indicated below each diagram. (**c**) Bar charts display the relative frequency of shared vs. unique ARGs within LIT and ENV for each species; *, *p* < 0.05; ****, *p* < 0.0001. (**d**) Boxplots depict Shannon diversity of ARG-associated taxa (ARGat), derived from in silico host mapping of ARGSs to putative microbial hosts, for LIT and ENV in both species; *, *p* < 0.05. Boxes show medians and interquartile ranges; whiskers denote 1.5× IQR

Cumulative ARG abundance was significantly higher in indoor environmental samples (ENV) than in fecal litter (LIT) (Fig. 2a). ARG read count in the LIT group proved to be 74,498, while the count in the chicken and duck farm environments group (ENV) was 78,2193; *p* < 0.0001 duck ENV: 42,746 vs. duck LIT: 41,341; *p* < 0.0001. Indoor ENV samples from duck farms accounted for 28% (42,746 out of 152,691) of all identified ARGs, whereas duck LIT samples contributed 27.08% (41,341 out of 152,691). In contrast, broiler LIT samples exhibited the lowest AMR levels, with a count of 33,157, representing 21.72% of all detected ARGs.

Additionally, ARG richness was assessed across LIT and ENV samples in both broiler and duck production systems (Fig. 2b). In broilers, ARGs shared between LIT and ENV samples accounted for 51.14% of all detected non-redundant ARGs (1,300/2,542); in ducks, the shared set represented 58.5% (1,459/2,494), indicating a substantial core resistome across matrices in both species.

Distinct rearing-specific unique ARG signatures were observed in both fecal litter and environmental samples. In the case of broiler chickens, unique resistances were present in nearly equal proportions in both fecal litter and indoor environments. However, the unique ARG in chicken reached 618 types, compared to 288 types in duck fecal litter, indicating that chicken fecal litter was nearly twice as heterogeneous as duck fecal litter.

In contrast, in farmhouse samples, ducks exhibited a substantially broader range of unique ARGs in the environment than in their fecal litter, with 747 unique types identified in duck farmhouse samples compared to 288 in duck. However, the unique ARG varieties in duck environmental samples were relatively similar to those observed in chicken indoor environmental samples, (chicken environment: 624 types, duck environment: 747 types).

The normalized abundance of rearing-system-specific ARGs (broiler vs. duck) was quantified across LIT, and ENV groups, and cross-habitat subsets (present in both sample types) (Fig. 2c).

Specifically, the relative frequency of the 618 unique resistances detected in chicken fecal litter was significantly higher (*p* < 0.02) than the relative frequency of the 624 unique resistances observed in the indoor environmental samples.

Nevertheless, in the case of ducks, significantly higher values were measured in the environment, which was also associated with a much larger number of resistance types (747 types, with a relative frequency of 0.0025) compared to the fecal litter samples (*p* < 0.0001), where a substantially smaller variety of resistance types was detected (288 types, with a relative frequency of 0.00045).

However, unique ARGs were negligible compared with the shared, cross-habitat ARG set detected in both fecal litter and the indoor environment for both rearing systems. The pooled abundance of shared ARGs across habitats was BC = 0.994 ± 0.00014 in broilers and DU = 0.995 ± 0.0041 in ducks (Fig. 2c). Within the shared category, habitat contributions were approximately proportional in both species (broilers: ENV 0.477, LIT 0.517; ducks: ENV 0.474, LIT 0.521), i.e., ~ 48:52 ENV: LIT in each case.

The diversity of ARG-associated taxa (ARGat) was assessed by in silico mapping of fecal litter- and indoor-derived ARGs to their putative microbial hosts, with comparisons stratified by habitat (LIT vs. ENV) and rearing system (broiler vs. duck) (Fig. 2d). This analysis revealed that Shannon diversity of ARGat was significantly higher in duck than in chicken samples in both fecal litter (LIT duck: 4.30 ± 0.37 vs. LIT broiler: 3.85 ± 0.58; *p* = 0.0010) and indoor environmental matrices (ENV duck: 4.36 ± 0.40 vs. ENV broiler: 4.03 ± 0.47; *p* = 0.020).

### Unique vs. shared ARGs in broiler chicken and duck farm indoor environments and their distribution across production phases

**Fig. 3:**
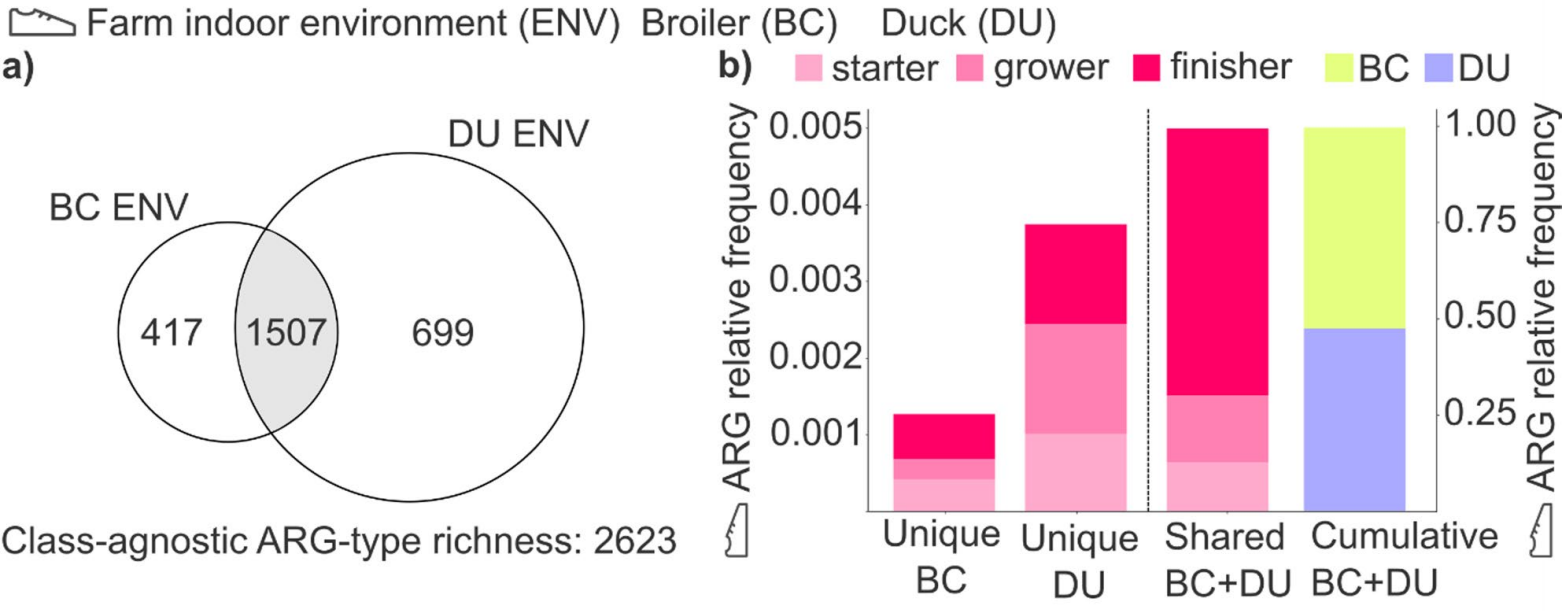
Shared and unique antibiotic resistance genes (ARGs) in broiler chicken and duck farm indoor environments: diversity, abundance, and dynamics across production phases. (**a**) Proportional Venn diagram showing the distribution of ARG types between broiler chicken (BC) and duck (DU) farm indoor environments (ENV). (**b**) Stacked bar charts represent the relative abundance of unique and shared ARG types in farm indoor environments across production phases (starter, grower, and finisher), and the cumulative relative frequency of ARGs in both systems

In farm indoor environmental samples (ENV), ARG-type richness (non-redundant) was comparable between broiler and duck systems (BC_ENV: 1,924; DU_ENV: 2,206, total across both: 2,623). In ENV samples, 1,924 non-redundant ARG types were detected for broilers and 2,206 for ducks. The analysis revealed that the ARGs shared between chicken and duck indoor environments were the most diverse, accounting for 1,507 types (57.5% of all ARG types). These shared ARGs were 2.16 times more diverse than those unique to duck environments (699 ARGs) and 3.61 times more heterogenic than those unique to chickens (417 ARGs) (Fig. 3a).

Additionally, duck ENV samples exhibited 1.67 times more unique ARG types compared to chicken farmhouse samples, with 699 types detected in ducks (representing 31.6% of the total ARG diversity in ducks) compared to 417 types in chickens (accounting for 21.7% of the total ARG diversity in chickens).

The load of environmental antimicrobial resistances was also investigated in both broiler- and duck-rearing systems (Fig. 3b). Stacked bar charts are used to illustrate the relative frequencies of unique indoor environmental ARGs in broilers and ducks, as well as those shared between the indoor environments of the two species across the production cycle (starter, grower, and finisher phases).

The shared cross-matrix subset of ARGs, present in the indoor environments of both broiler and duck systems, displayed markedly higher relative abundance (0.995; *p* < 0.0001) compared with unique ARGs (0.005). The abundance of shared ARGs increased progressively across production, with the lowest values in the starter phase (0.130) and the highest in the finisher phase (0.695; *p* < 0.0001).

Although unique ARGs occurred at very low frequencies, clear species-specific differences were observed (broiler: 0.0013 vs. duck: 0.0037). In ducks, the highest values were observed in the grower phase (0.0014) and the lowest in the starter phase (0.0010), with finisher levels intermediate (0.0013). In broilers, the lowest unique-ARG frequency was recorded in the grower phase (0.00026), while starter (0.00042) and finisher (0.00058) phases showed comparable levels. In ducks, unique ARGs were proportionally distributed across the three growth phases.

### Distribution of the most prevalent antibiotic resistance classes in broiler and duck poultry farms

**Fig. 4:**
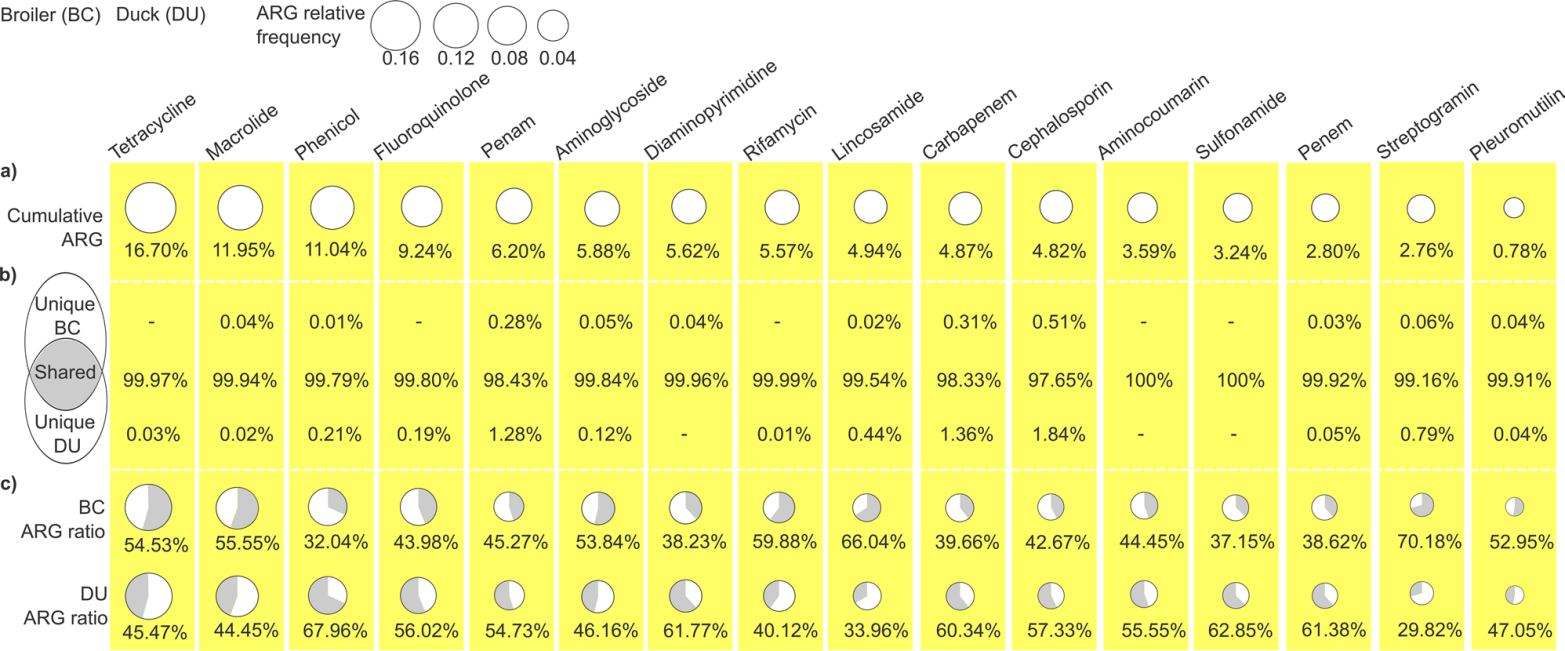
Relative frequencies of poultry antibiotic resistance genes (ARGs) summarized by antibiotic class, with within-class partitioning into broiler-unique, duck-unique, and shared subsets. (**a**) Bubble plot of the cumulative relative frequency of ARGs across the top 16 antibiotic classes; bubble area is proportional to cumulative relative frequency. (**b**) Venn diagram partitioning ARGs within these classes into broiler-unique (unique BC), duck-unique (unique DU), and shared sets; the accompanying table reports their class-wise relative frequencies. (**c**) For each class, the embedded pie chart indicates the within-class contribution of broiler (BC)- vs duck (DU)-derived ARGs

The most prevalent antimicrobial resistance classes were identified by ordering ARGs by normalized relative frequency and mapping each gene to its corresponding class; class-level prevalence was defined as the cumulative relative frequency of the constituent ARGs. (Fig. 4).

Tetracycline was identified as the most prevalent resistance class, with a relative frequency of 16.70%, followed by macrolides (11.95%), phenicols (11.04%), fluoroquinolones (9.24%), and other resistance classes such as penam (6.20%), aminoglycoside (5.88%), diaminopyrimidine (5.62%), rifamycin (5.57%), lincosamide (4.94%), carbapenem (4.87%), cephalosporin (4.82%), aminocoumarin (3.59%), sulfonamide (3.24%), penem (2.80%), streptogramin (2.76%) and pleuromutilin (0.78%) (Fig. 4a).

To evaluate host-associated structure, we assessed how cumulative ARGs across the top 16 ARG classes partitioned between broiler and duck indoor environments. In nearly all classes, the overwhelming majority consisted of shared ARGs (99.51% ± 0.73%), while uniquely detected resistances were rare (0.24% ± 0.45%). In several classes, including aminocoumarins and sulfonamides, no unique ARGs were detected at all. Species-specific patterns were also evident: in broilers, tetracycline, fluoroquinolone, and rifamycin classes lacked unique resistances, whereas in ducks, no unique ARGs were detected for diaminopyrimidines (Fig. 4b).

Among shared resistances, several classes were more prevalent in broilers (tetracycline, macrolides, aminoglycosides, rifamycin, lincosamides, streptogramins, pleuromutilins), with streptogramins showing the largest difference (2.35× higher in broilers). In contrast, the remaining classes were more dominant in ducks (phenicol, fluoroquinoline, penam, diaminopyrimidine, carbapenem, cephalosporin, aminocoumarin, sulfonamide, penem), with phenicols being 2.12× higher in ducks (Fig. 4c).

### Identification of above-average antimicrobial resistances (aaARGs) of broiler chicken and duck farm environments and their associated key-carrier species

**Fig. 5:**
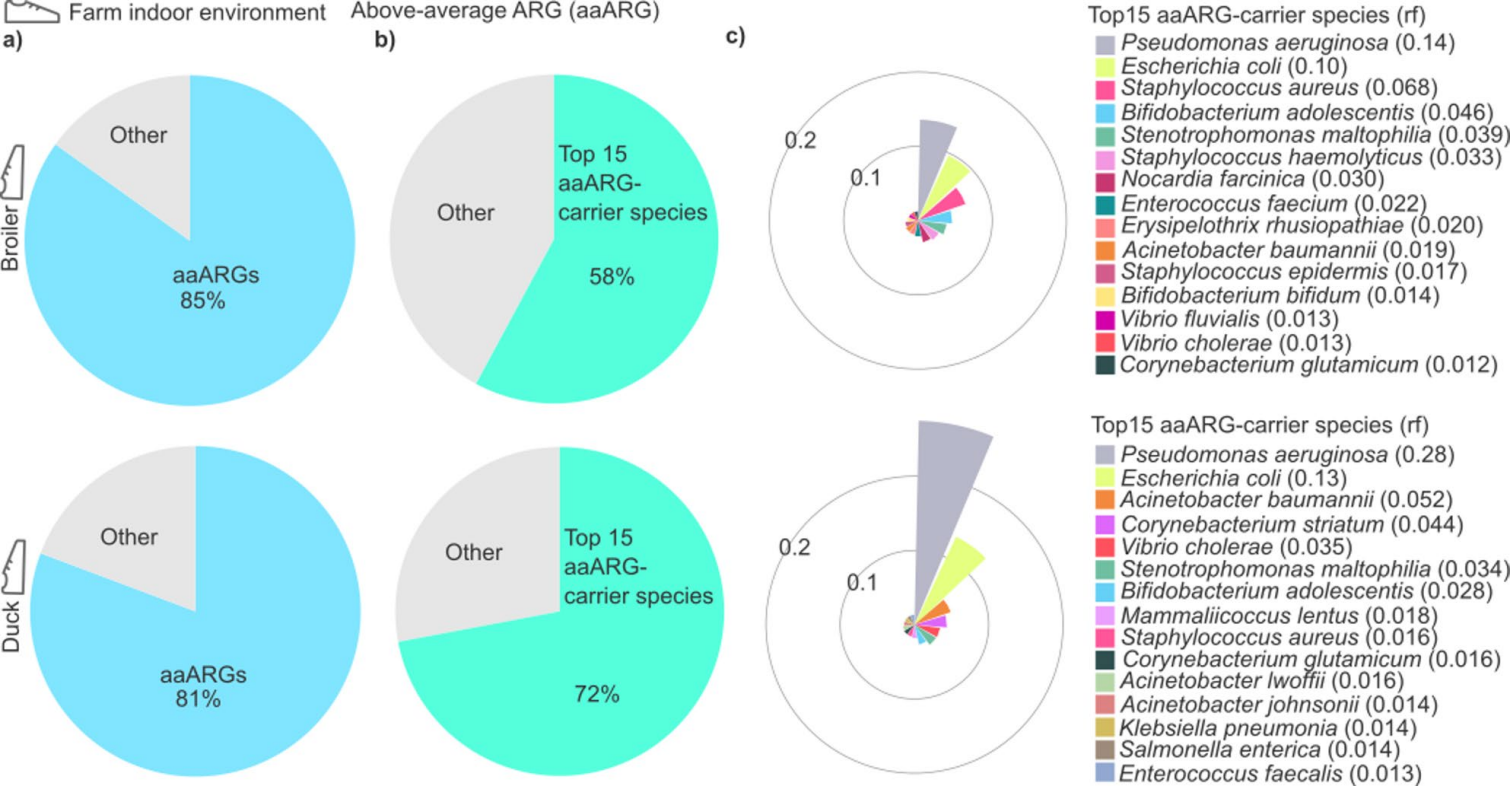
Distribution and key contributors of above-average antimicrobial resistances (aaARGs) in broiler and duck farm indoor environments. (**a**) Pie charts show the distribution of aaARGs within the resistome for broiler and duck (slices = aaARG relative abundance). (**b**) Pie charts show the contribution of the top 15 aaARG-carrier species to the aaARG profiles in broiler and duck (slices = aaARG-carrier species relative abundance). (**c**) Polar plots depict species-level relative frequencies for top15 aaARG-carrier species; color legend at right

We placed specific emphasis on above-average ARGs (aaARGs). While low-abundance ARGs can be contextually important, very low-frequency detections are more likely to reflect background signal; therefore, aaARGs, defined as ARGs exceeding the within-host mean relative frequency in broiler or duck indoor resistomes were prioritised for comparative analyses. Using this definition, broiler indoor resistomes showed a slightly higher mean ARG relative frequency (0.00052) than ducks (0.00045) when normalised to the total resistome (Fig. 5a). Based on our findings, the distribution of aaARGs in the poultry farm indoor environment was highly prevalent in both broiler chicken and duck farming systems, with an average prevalence of 83% ± 2.8%.

Subsequently, the top15 aaARG-carrier species, ranked by species-level relative abundance were identified and subjected to comprehensive in silico analysis in broiler and duck farm environments (Fig. 5b). These species accounted for 58% of total resistance in broiler chickens and 72% in ducks.

The relative abundances of the top15 aaAMR-carrier species were assessed across samples (Fig. 5c). *Pseudomonas aeruginosa* and *Escherichia coli* were identified as the dominant contributors in both broiler and duck farm indoor environments. Notably, *Pseudomonas aeruginosa* exhibited a relative frequency in ducks that was double that observed in broilers (*Pseudomonas aeruginosa* duck relative frequency: 0.28; broiler: 0.14). Similarly, *Escherichia coli* showed a stronger association with ducks compared to broilers (*Escherichia coli* duck relative frequency: 0.13; chicken: 0.10). In addition to these findings, other prominent top15 aaARG-carrier species in ducks included *Acinetobacter baumannii* (relative frequency: 0.052) and *Corynebacterium striatum* (relative frequency: 0.044). Conversely, in chickens, significant aaARG-carrier species included *Staphylococcus aureus* (relative frequency: 0.068) and *Bifidobacterium adolescentis* (relative frequency: 0.046).

### Broiler- and duck-specific microbial fingerprints identified from the distribution of key aaARG carrier species in farm environments

**Fig. 6:**
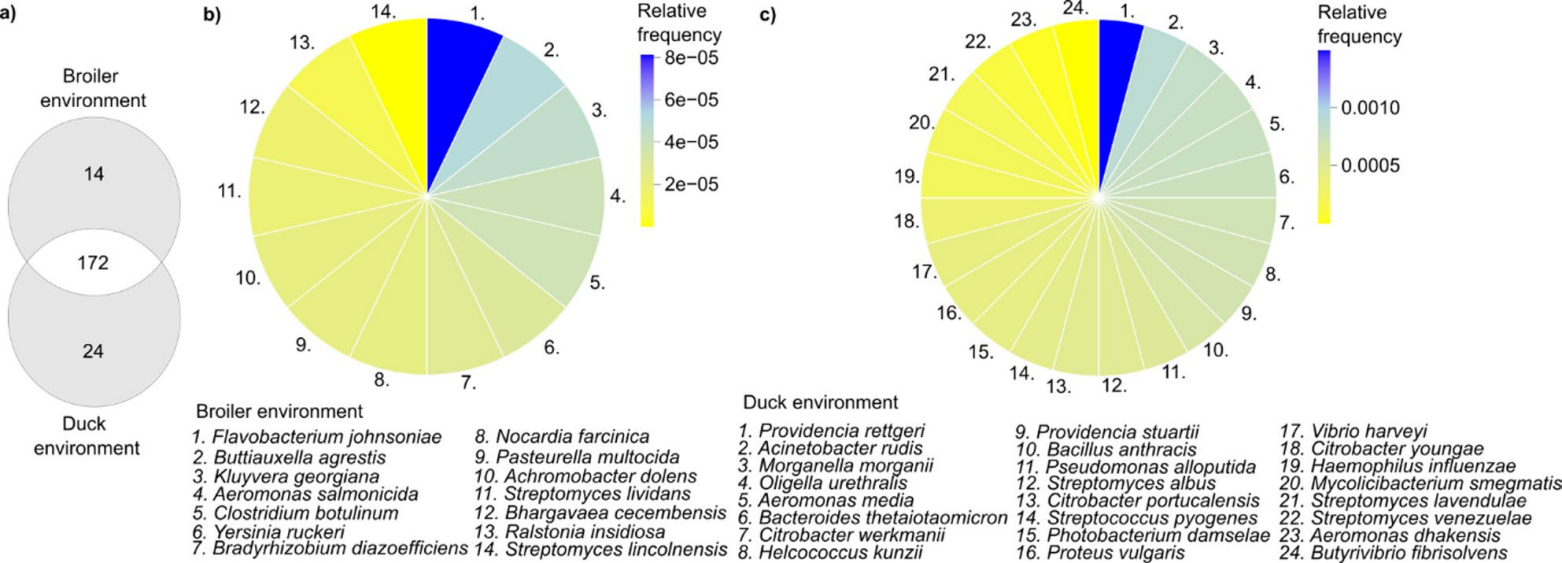
Identification of broiler- and duck-unique microbial fingerprints of key above-average antimicrobial resistance (aaARG) carrier species in poultry farm environments. (**a**) Venn diagram illustrating the distribution of aaARG-carrier species between broiler and duck farm environments. Circular heatmaps of key aaARG-carrier species in broiler (**b**) and duck (**c**) farm environments, the color representing the relative frequencies. Meaning the numbers are listed above, in both broiler chicken and duck farm environments

A total of 172 aaARG carrier species were identified as cross-habitat taxa shared between broiler chicken and duck farm indoor environments (Fig. 6a).

Additionally, 14 aaARG-carrier species were detected exclusively in broiler indoor environments, whereas 24 were unique to duck indoor environment. The within-environment relative abundances of broiler-unique and duck-unique aaARG-carrier species are shown for broiler (Fig. 6b) and duck (Fig. 6c) settings, respectively.

Notably, *Providencia rettgeri* was the only uniquely detected species in the duck environment with a relative abundance exceeding 0.001 (*Providencia rettgeri* relative frequency: 0.0015). Our analysis also highlighted several pathogens of dual significance for animal production and human health within these environments. In chicken-specific settings, the Gram-negative *Pasteurella multocida* was uniquely identified and is known for causing fowl cholera, a highly contagious and economically devastating disease in poultry [27, 28]. Additionally, *Clostridium botulinum*, an anaerobic spore-forming, toxinogene bacterium, was detected. Its toxin is among the most potent neurotoxins known and represents a serious threat to human health, particularly through foodborne exposure [29]. In duck-specific environments, *Bacillus anthracis* was uniquely identified. Although it is not typically associated with poultry, its spores can be ingested through contaminated soil or feed, potentially leading to localized outbreaks in ducks [30, 31]. Anthrax, caused by *B. anthracis*, is a zoonotic disease with significant public health implications [32]. Another pathogen identified in duck environments was *Haemophilus influenzae*, a bacterium primarily associated with human respiratory infections, meningitis, and otitis media [33]. Its detection in poultry-associated environments raises concerns regarding possible cross-species transmission. Finally, *Streptococcus pyogenes* was detected in duck-specific environments, with its presence underscoring potential zoonotic risks [34].

### Cross-habitat aaARG-carrier species across broiler, duck, and indoor environments

**Fig. 7:**
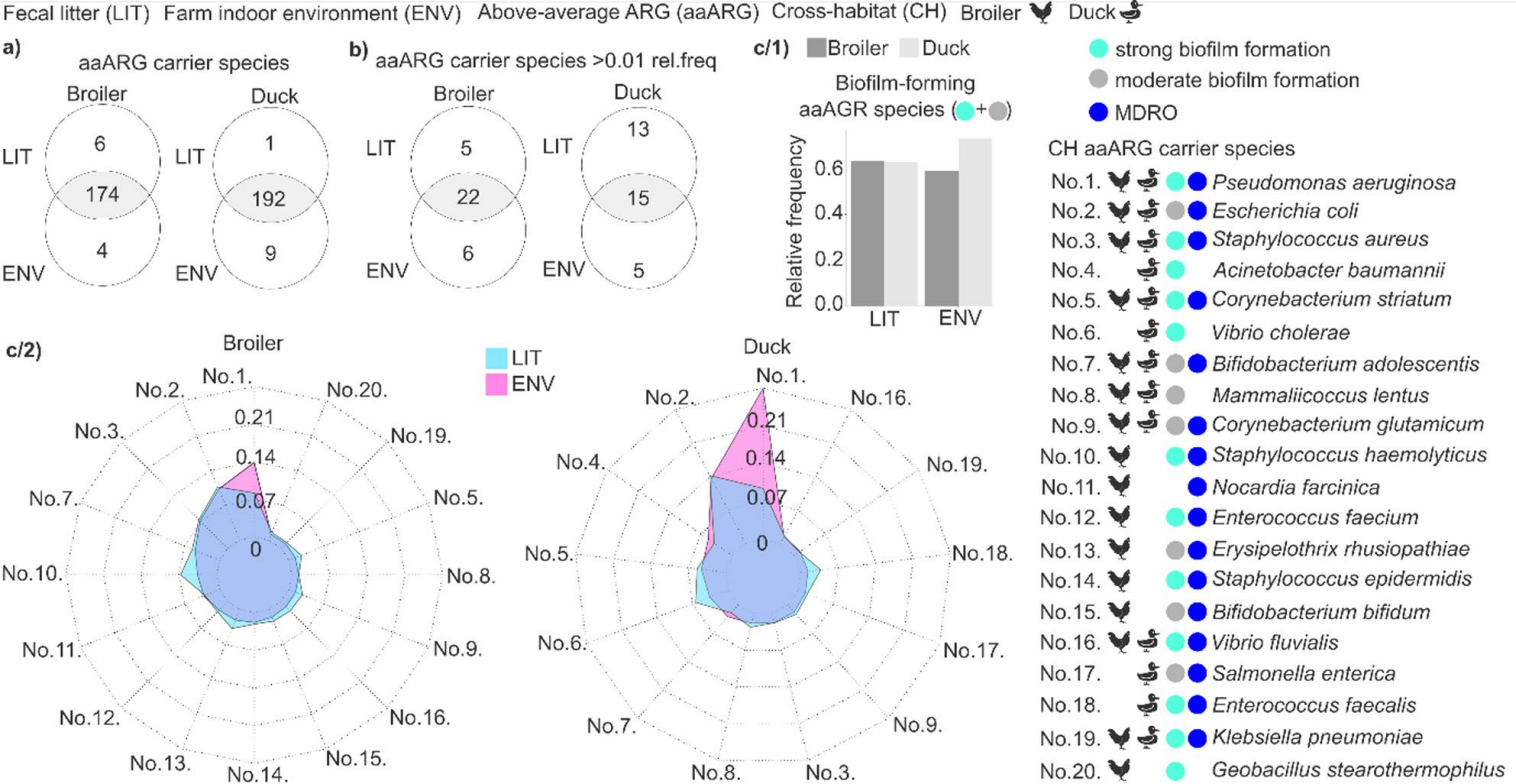
Identification of cross-habitat above-average antimicrobial resistances carrier species (CH aaARGs) across broiler, duck, and indoor environments. (**a**) Venn diagrams illustrating the overlap of aaARG carrier species between fecal litter (LIT) and farm indoor environmental (ENV) samples in both duck and broiler chicken production systems. (**b**) Subset analysis of high transmission potential aaARG carrier species exceeding 0.01% relative abundance. (**c**/1) Bar plot shows the cumulative relative frequency of the cross-habitat aaARG (CH aaARG) carrier biofilm-forming species in LIT and ENV samples, between broiler and duck. (**c**/2) Radar plots displaying the relative abundance distribution of CH aaARG carrier species between LIT and ENV samples for broiler chickens and ducks. Broiler chicken and duck icons indicate the presence of CH aaARG carrier species, distinguishing those found in both rearing systems from those exclusive to either the broiler or duck systems. The identified CH aaARG carrier species exceeding 0.01% relative abundance were also classified based on their biofilm-forming capacity (grey for moderate and light blue for strong) and multidrug resistance (resistance to more than three antibiotics; dark blue)

The extent of potential resistance exchange within poultry reservoirs was assessed by identifying cross-habitat aaARG carrier species (CH aaARG), defined by shared occurrence in flocks and their matched farm indoor environments.

A significant overlap of key aaARG carrier species was observed across fecal litter (LIT) and indoor samples (ENV) (Fig. 7a). Specifically, in broilers, 174 aaARG carrier species (94.57% of all detected species) were consistently observed across both LITand ENV samples; in ducks, 192 aaARG carriers (95.05%) were identified.

A more stringent analysis was applied by restricting inclusion to aaARG carrier species detected in both LIT and ENV samples with relative abundances > 0.01. Based on their elevated abundance and transmission potential, these species were classified as high-transmission-potential aaARG carrier species (Fig. 7b) (Supplementary File 1).

In broiler environments, 22 of 33 (66.7%) high-transmission-potential aaARG carriers were shared between LIT and ENV samples, whereas in duck environments, only 15 of 33 (45.5%) species were common to both habitats.

The number of unique, habitat-specific aaARG carrier species was 1.2 times higher in broiler environments, whereas in duck environments, unique species accounted for 15.1% in ENV samples and 39.4% in LIT samples.

Within the set of cross-habitat aaARG carriers, the distribution of biofilm-forming taxa, classified into moderate and high biofilm-forming capacity based on mechanistic evidence (Supplementary File 2), together with multidrug-resistant organisms (MDROs) was analysed across LIT and ENV samples (Fig. 7c).

Across biofilm-forming aaARG carrier species, cumulative occurrence was similar in LIT samples between rearing systems, whereas higher occurrence was observed in ENV samples from duck houses relative to broiler houses; however, this difference did not reach statistical significance (Fig. 7c/1).

Among biofilm-forming and/or MDRO taxa, 20 distinct CH aaARG carriers were identified. Of these, nine were present in both poultry species (No. 1: *Pseudomonas aeruginosa*, No. 2: *Escherichia coli*, No. 3: *Staphylococcus aureus*, No. 5: *Corynebacterium striatum*, No. 7: *Bifidobacterium adolescentis*, No. 8: *Mammaliicoccus lentus*, No. 9: *Corynebacterium glutamicum*, No. 16: *Vibrio fluvialis* and No. 19: *Klebsiella pneumoniae*). Sixteen CH aaARG carriers were detected in broilers (Fig. 7c/2) and thirteen in ducks (Fig. 7c/3), indicating lower diversity in duck production systems.

Across both broiler chicken and duck rearing systems, *Pseudomonas aeruginosa* displayed a notably higher prevalence in ENV samples in both bird species (ENV *Pseudomonas aeruginosa* duck rel. freq.: 0.29 vs. duck LIT: 0.094; ENV chicken: 0.14 vs. LIT chicken: 0.082).

Despite these variations, *Escherichia coli* remained consistently abundant across both LIT and ENV samples in both rearing systems.

In duck, *Enterococcus faecalis* and *Vibrio cholerae* were more prevalent in LIT samples (LIT *E. faecalis* rel. freq.: 0.038 vs. ENV: 0.014; LIT *V. cholerae* rel. freq.: 0.063 vs. ENV: 0.036), while in chickens, *Corynebacterium striatum* followed a similar trend (LIT *C. striatum* rel. freq.: 0.025, ENV: 0.012).

Furthermore, in broiler chickens, *Erysipelothrix rhusiopathiae* (LIT rel. freq.: 0.038, ENV: 0.020), *Vibrio fluvialis* (LIT rel. freq.: 0.024, ENV: 0.013), *Corynebacterium glutamicum* (LIT rel. freq.: 0.027, ENV: 0.013) showed a higher prevalence in biological samples. Similarly, in ducks, *Mammaliicoccus lentus* (LIT rel. freq.: 0.026, ENV: 0.018) followed the same pattern.

### Differentially abundant aaARGs and carrier species in AB-free and AB-treated farm indoor environments

**Fig. 8:**
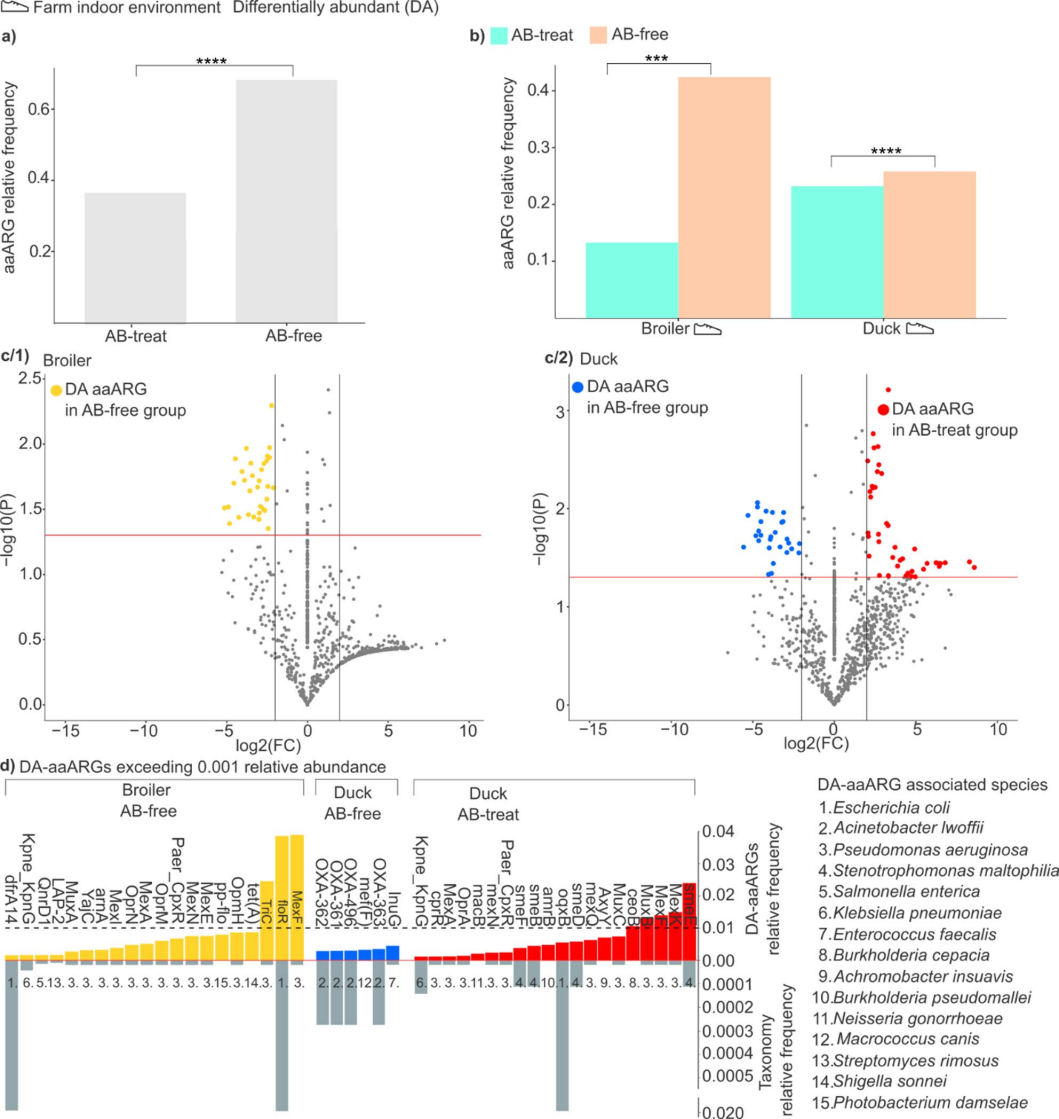
Comparison of above-average antimicrobial resistance (aaARGs) and their carriers between antibiotic-free (AB-free) and antibiotic-treated (AB-treat) farm indoor environments in broiler chickens and ducks. (**a**) The cumulative distribution of above-average antimicrobial resistances (aaARGs) in antibiotic-treated (AB-treat) and antibiotic-free (AB-free) environments across both broiler chicken and duck-rearing systems. Statistical significance was denoted with asterisks (****p < 0.0001). (**b**) Rearing-specific comparison of aaARG relative frequencies in AB-treated and AB-free environments. Statistical significances were denoted with asterisks (***p < 0.001, ****p < 0.0001). (**c**/1) Volcano plots depicting differentially abundant aaARGs (DA-aaARGs) in chicken AB-free farm indoor environments compared to AB-treated farm environment. Microorganisms depicted by yellow dots are significantly more abundant in AB-free environments (p-value < 0.05; log2 fold change < -2). (**c**/2) In duck samples, species significantly enriched in AB-free environments are shown as blue dots (p-value < 0.05; log2 fold change < -2). In contrast, species significantly enriched in AB-treated environments are shown as red dots (p value < 0.05, log2 fold change > 2). (**d**) Barplots represent the DA-aaARG, highlighted by volcano analysis. The upward-facing barplots show the DA-aaARGs relative frequency in increasing order of relative frequency > 0.001. The dashed black line represents the 0.01 relative frequency. The downward-facing bars represent the relative frequency of species related to DA-aaARGs

The accumulation of aaARGs was assessed in antibiotic-treated (AB-treat) and antibiotic-free (AB-free) environments across both broiler chicken and duck-rearing systems (Fig. 8a). Our analysis revealed a significantly lower relative frequency of aaARGs in AB-treated poultry environments.

Subsequently, a rearing-specific analysis on aaARG relative frequencies in AB-treated and AB-free environments was conducted (Fig. 8b). Interestingly, in both broiler chicken and duck farm indoor environments, aaARG frequencies proved to be significantly lower (*p* < 0.05) in AB-treated settings than in AB-free environments.

Furthermore, aaARGs showing significant changes in normalized relative abundance between AB-free and AB-treated environments were designated and referred to as differentially abundant aaARGs (DA-aaARGs) (Fig. 8c).

Within poultry farm indoor environments, 60 DA-aaARGs were identified, 32 in broiler (53.3%) (Fig. 8c**/1**) and 28 in duck systems (46.7%) (Fig. 8c**/2**). Next, only differentially abundant aaARGs with relative frequency > 0.001 (DA-aaARGs RF ≥ 0.001%) were retained; features below this threshold were excluded, the associated carrier species were identified, and their relative frequencies were quantified (Fig. 8d).

In broiler indoor environments, 32 DA-aaARGs were identified; among those overrepresented under AB-free versus AB-treated conditions, 20 exhibited relative frequency ≥ 0.001 (Fig. 8d). *Escherichia coli*, carrying *floR* and *dfrA14*, was identified as the most prevalent DA-aaARGs carrier (mean relative frequency = 0.020) in antibiotic-free environments.

In ducks, six DA-aaARGs were overrepresented in antibiotic-free environments at relative frequencies > 0.001; most were predominantly associated with *Acinetobacter lwoffii* (*OXA-363*,* OXA-496*,* OXA-361*,* OXA-362*). Furthermore, 20 aaARGs were identified as significantly overrepresented in the antibiotic-treated group. The most abundant was *smeE* (associated with *Stenotrophomonas maltophilia*; rel. freq. ≈ 1.1 × 10⁻⁴), followed by *mexK*, *mexF*, and *muxB* (linked to *Pseudomonas aeruginosa*; ≈ 1.8 × 10⁻⁵), and *ceoB* (*Burkholderia cepacia*; ≈ 1.2 × 10⁻⁸). Notably, mirroring broiler environments, *Escherichia coli* was identified as the most prevalent differentially abundant species in duck samples (rel. freq. = 0.020).

### Newly acquired resistances in broiler chicken and duck farm indoor environments

**Fig. 9:**
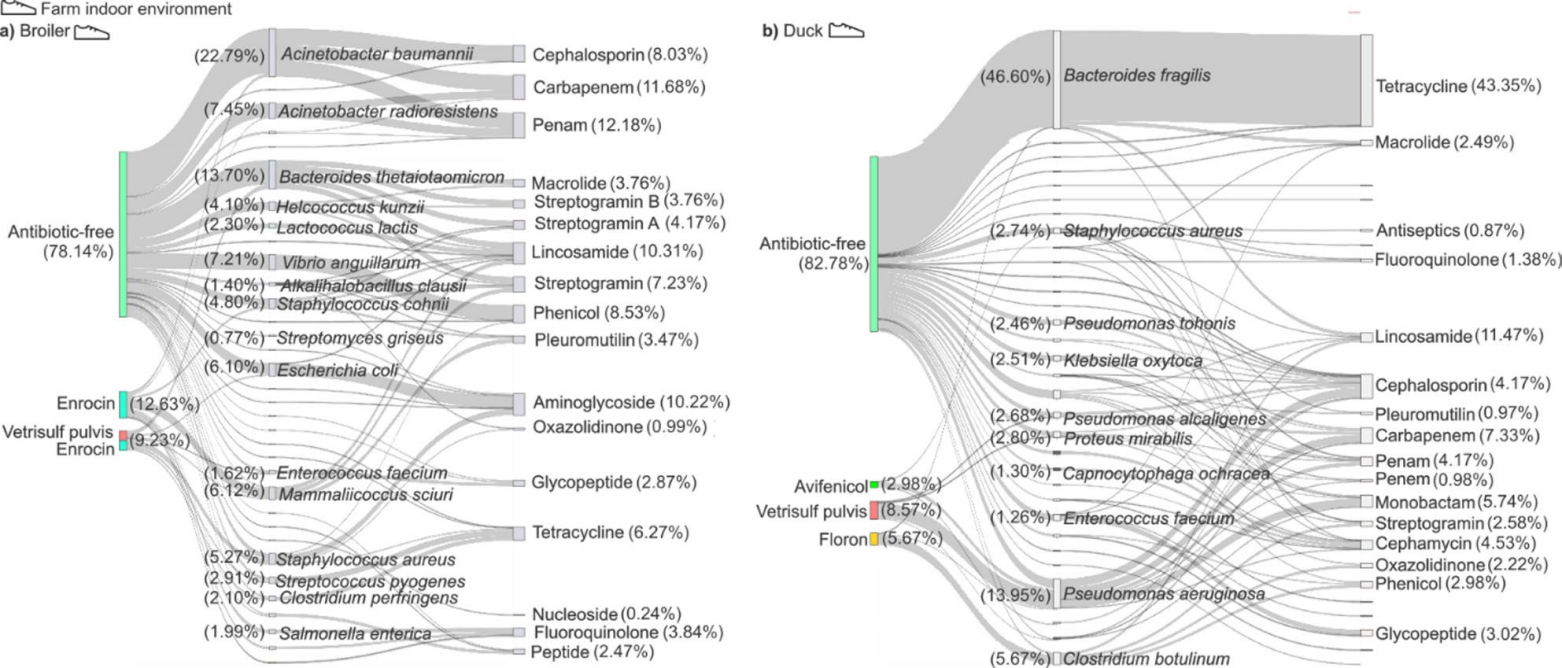
Sankey Diagram of newly acquired environmental resistances (NAeRs) distribution in broiler chicken and duck farm indoor environments. (**a**) Sankey diagram illustrating the distribution of newly acquired environmental resistances (NAeRs, resistances that appear during the growth of the animal) in broiler chicken farm indoor environments. The diagram highlights the associations between antibiotic treatments (AB-free environments, and environments of flocks treated with Enrocin and Vertisulf Pulvis + Enrocin), key ARG carrier species (middle block), and their corresponding resistance categories (rightmost block). The thickness of the lines is proportional to the distribution. (**b**) Sankey diagram showing the distribution of NAeRs in duck farm indoor environments. The diagram visualizes the relationship between antibiotic treatments (AB-free environments, and environments of flocks treated with Avifenicol, Vertisulf Pulvis, and Floron), key ARG carrier species (middle block), and their associated resistance categories (rightmost block). The thickness of the lines is determined by the distribution

The emergence of newly acquired environmental ARGs (NAeRs) was assessed in broiler and duck farm indoor environments under antibiotic-free (AB-free) and prophylactically antibiotic-treated (AB-treated) conditions, with NAeRs first detected exclusively during the finisher phase (Fig. 9).

Broiler production cycles operated under AB-free conditions and cycles managed with flock-level prophylactic antibiotic regimens (Enrocin, Vertisulf Pulvis, or both) were included. (Fig. 9a). Among broiler chickens, the majority of NAeRs (78.14%) were detected in AB-free farm indoor environments, while a smaller proportion (12.63%) was linked to Enrocin-treated environments, and an even lower fraction (9.23%) was associated with environments exposed to combined Enrocin and Vertisulf pulvis treatments. The most frequently identified newly acquired environmental resistance-associated species in broiler chickens was *Acinetobacter baumannii* linked to resistance in three major antimicrobial classes: penams (32.86%), carbapenems (34.28%), and cephalosporins (32.86%). The second most prominent NAeR carrier was *Bacteroides thetaiotaomicron*, which was primarily associated with AB-free environments (11.50%), although a smaller subset of NAeRs (2.19%) was also detected in Vertisulf pulvis plus Enrocin combination-treated environments. The resistance profile of *B. thetaiotaomicron* was highly diverse, with streptogramin (20%), macrolide (20%) and lincosamide resistances (20%).

In duck farm environments, the majority (46.60%) of NAeRs were linked to *Bacteroides fragilis*, with an overwhelming 98.9% of these resistances detected in AB-free environments (Fig. 9b). Among the identified NAeRs associated with *B. fragilis*, tetracycline resistance was the most prevalent, accounting for 91.74%. Additionally, *B. fragilis* was also linked, albeit in smaller proportions, to macrolide (3.90%) and lincosamide (3.90%) resistances. The second most common NAeR carrier species in duck farm indoor environments was *Pseudomonas aeruginosa*, which was predominantly identified in Vertisulf Pulvis-treated (6.96%) and AB-free flock environments (4.34%). NAeRs carried by *Pseudomonas aeruginosa* exhibited remarkable diversity, with cephalosporin resistance being the most prevalent (29.38%), followed by monobactam (27.92%), carbapenem (26.76%), and penam resistances (13.22%). The third most frequent NAeR carrier species was *Clostridium botulinum*, which was primarily detected in Floron-treated environments (4.53%), though it was also present in smaller proportions in AB-free settings (1.14%). NAeRs associated with *C. botulinum* exhibited an exceptionally diverse resistance profile, encompassing lincosamide, streptogramin, oxazolidinone, and phenicol resistances (all with 25.0% proportion).

## Discussion

An escalation of antimicrobial resistance is recognized globally, with intensive poultry production implicated in the emergence and dissemination of resistance [3].

Despite mitigation efforts, antibiotics are still administered at the flock level, including for prophylaxis, thereby imposing selective pressure on farm environment resistomes with implications for both animal and human health [3, 5].

Excreted resistant microbes and ARGs constitute a major on-farm reservoir that can disseminate via aerosolization, runoff, and personnel- or fomite-mediated transfer, thereby amplifying horizontal gene transfer and sustaining transmission within and beyond farms highlighting the need for more detailed characterization of such reservoirs and their dynamics [9–13].

Therefore, we aimed to apply parallel shotgun metagenomics to poultry manure (fecal litter) and indoor environmental samples to enable a system-level comparison that identifies whether manure or the indoor environment is the dominant reservoir, quantifies host–environment coupling, describes the diversity and structural architecture of the resistome, and tracks stage-resolved temporal dynamics across the production cycle.A two-year longitudinal study was conducted under real-world, commercial-scale conditions in intensive broiler and duck production systems in Hungary. Husbandry, feeding, and infection-control were harmonized across sites to maximize representativeness and comparability.

A dual sampling strategy was implemented across multiple independent production cycles to simultaneously profile host-associated and environmental compartments.

Both broilers and ducks were housed in temperature-controlled facilities under industry-standard rearing conditions. The production pipeline was standardized across systems into three two-week stages (starter, grower, finisher) to align husbandry and sampling windows.

Temporal resolution was enhanced by daily monitoring throughout one complete cycle in each system, supporting attribution of resistome dynamics to stage-linked and environment-linked drivers rather than episodic perturbations.

Based on our observations, across the aggregated shotgun metagenomic dataset, antimicrobial-resistance-gene prevalence proved to be significantly higher in indoor farm environment samples than in fecal litter. The significance of this finding lies in the observation that an environmental reservoir enriched in antimicrobial-resistance genes serves as a proximal source of flock re-contamination [35].

Also, a significant divergence in reservoir dominance was identified, with indoor environmental serving as the principal ARG reservoir in duck operations, in contrast to fecal litters serving as the major reservoir in broiler operations. This finding is particularly noteworthy given that both systems were operated in fully enclosed houses.

Overall, ARG diversity was similar between duck and broiler production systems. However, within duck production, environmental samples contained approximately 2.6-fold more unique ARG types than corresponding fecal litter samples. In addition, comparison of environmental samples across production systems revealed a modestly higher ARG-type richness in duck environments compared with broiler environments (1.15-fold increase).

In line with our theory, a plausible explanation is that wet, high-humidity microenvironments in duck houses promote biofilm formation and the persistence of water-associated opportunists; once established, these biofilms can continuously release cells and DNA fragments into aerosols and redeposit onto fomites, effectively amplifying the environmental ARG reservoir [36].

By contrast, broiler facilities more commonly employ closed nipple drinkers and maintain comparatively drier litter, such that ARGs are preferentially retained within fecal dropping manure and its dust fraction [37].

This interpretation is also consistent with prior reports implicating waterfowl systems in disproportionate environmental resistance loading and documenting abundant ARG-bearing microbial aerosols in duck houses as efficient dissemination vehicles [38–40]. Additional, non-exclusive mechanisms that could further reinforce the observed enrichment include biofilm-facilitated horizontal gene transfer that broadens ARG repertoires in wet microhabitats [22] and co-selection by metal residues, which can be particularly effective within biofilm matrices [41]. Supporting the latter, our previous work showed that duck feed contained ~ 1.03-fold higher Zn and ~ 1.2-fold higher Cu than broiler feed, which could plausibly contribute to co-selection and cross-resistance dynamics in the duck production environment [42].

The near-equality of ARG diversity across broiler and duck production might be readily accounted for by a shared pan-resistome circulating through common inputs and infrastructures; by disinfectant-mediated co-selection that sustains mobile elements and multidrug mechanisms within biofilms [43].

The substantially higher Shannon diversity of resistance-carrier species observed in duck systems, across both fecal litter and indoor matrices signals a broader ecological host range and functional redundancy, conditions that favor persistence, horizontal gene transfer, and aerosol- or fomite-mediated spread [44].

The shared environmental ARG pool between the two rearing systems indoor environment accounted for 57.5% of all identified ARG types suggesting a high potential for cross-species transmission.

Even under strict cleaning and disinfection (C&D) protocols, the farm environment can remain an effective ARG reservoir [45, 46].

In our data, environmental AMR/ARG load accumulated steadily throughout the grow-out, reaching its highest levels in the finisher phase just before depopulation. This is concerning, as the reservoir is largest at the point when dust and aerosols are most easily mobilized, raising the risk of re-contamination and carryover. The pattern can be partly explained by continuous reseeding of the environment during production: flock shedding (feces, dander), aerosolized dust, and waterline back-contamination progressively accumulate on litter, fixtures, and drinker systems, raising background loads even in the absence of clinical disease.

Some antimicrobial resistances have limited clinical or epidemiological impact, especially when they occur at low frequencies. In contrast, dominant resistances often exhibit greater adaptability, allowing them to persist across different environments and hosts, thereby increasing their potential for dissemination within farm settings, food chains, and even clinical environments [47].

Antibiotic treatments were administered in 37.5% of production cycles in broiler chickens and 42.9% in ducks, highlighting a comparable reliance on interventions across both rearing systems, aligning with Hungary’s moderate veterinary antibiotic usage relative to other European countries [48].

Ducks were treated with sulfonamides, diaminopyrimidines, and fluoroquinolones, whereas broilers received fluoroquinolones, phenicols, sulfonamides, and diaminopyrimidines. The prominence of phenicol resistance in ducks despite no direct phenicol treatment is plausibly explained by co-selection and genetic linkage: phenicol resistance determinants frequently co-occur on mobile genetic elements with sulfonamide/trimethoprim and quinolone resistances and with metal-resistance loci and are efficiently maintained in moisture-stable biofilms [49].

In our dataset, several production cycles were completed without antimicrobial use, reflecting routine commercial practice in which antibiotic-free production is feasible but not universal. This variation allowed comparisons between AMU and non-AMU cycles within the same production system. Similar patterns have been reported in the EU: in Germany, 31.2% of broiler flocks received no antimicrobials during the production cycle [50]; across Europe, AMU shows wide between-farm and between-country heterogeneity; and specific technologies (e.g., on-farm hatching) can markedly raise the proportion of antimicrobial-free flocks (≈ 48% vs. 12% with conventional hatching in Belgium) [51]. Moreover, EU-level surveillance documents a sustained contraction in veterinary antibiotic sales (53% decrease from 2011 to 2022), even though official monitoring emphasizes mg/PCU sales/use rather than reporting the share of “antibiotic-free” flocks per se [52].

By focusing on above-average resistances, we prioritize the most significant resistance mechanisms, those most likely to be exchanged between animals and the environment. While total ARG read counts were significantly higher in duck environments, the mean relative frequency of above-average ARGs was higher in broiler chickens, likely reflecting a greater proportion of low-frequency resistance markers in ducks.

The environmental presence of key aaARG-carrying species in poultry farming raises significant concerns about the potential for cross-species transmission and subsequent human infections. Our findings highlight *Pseudomonas aeruginosa* and *Escherichia coli* as the most prominent high-resistance carrier species, both of which are well-documented human pathogens frequently also implicated in healthcare-associated infections [53]. Additionally, the detection of other significant resistance carrier species, including *Acinetobacter baumannii* (a major cause of ventilator-associated pneumonia), *Corynebacterium striatum*, *Staphylococcus aureus* (a leading pathogen in bloodstream infections), and *Stenotrophomonas maltophilia*, further amplifies concerns [54–57].

Notably, *Providencia rettgeri* was uniquely identified in duck farm environments, which is known for its multidrug-resistant traits, particularly against carbapenems and aminoglycosides [58]. Similarly, the detection of *Bacillus anthracis* in duck farm environments is concerning, as its highly resilient spores can persist long-term in soil and feed, posing a significant zoonotic threat [59]. Furthermore, human-associated bacterial species such as *Haemophilus influenzae* and *Streptococcus pyogenes* were also detected in duck farm environments indicating potential human-to-animal bacterial exchange, likely via farmworkers, contaminated feed, or water sources.

The identification of *Pasteurella multocida*, the primary causative agent of fowl cholera [28], and *Clostridium botulinum*, a neurotoxin-producing, spore-forming bacterium [29], in broiler chickens highlights their significant economic and health implications in poultry production, emphasizing the need for strict biosecurity and hygiene management to prevent disease outbreaks, contamination, and potential foodborne transmission.

Interestingly, while only 51.14% of ARGs were shared between biological and environmental samples in broiler chickens and 58.5% in ducks, the overlap among above-average resistance-associated species was remarkably high, reaching 95% in both poultry species.

Refining our analysis to aaARG carrier species with high-transmission potential (≥ 0.01 relative frequency with aaARGs) yielded 37 species and revealed asymmetric host-environment coupling. In broilers, 66.67% of cross-habitat high-risk carriers were detected in both manure and environmental samples, indicating a tightly coupled source-sink system with efficient two-way ARG movement. In ducks, the overlap was 45.45%, consistent with stronger partitioning between compartments; notably, 39.4% of habitat-unique aaARG carrier species with high-transmission potential were confined to manure, an intriguing counterpoint given that ducks showed significantly higher overall ARG prevalence in the environment. Thus, while the duck environmental reservoir is ARG-rich, the manure preferentially harbors a distinct subset of high-transmission aaARG carriers. Consequently, the ability of biofilm-forming bacteria poses a major challenge for infection control and biosecurity measures in poultry production systems [22, 60].

In the case of broiler chickens, among the high-transmission potential cross-habitat aaARG carrier species detected in both fecal litter and indoor farm environments, several biofilm-forming bacteria were identified, including *Escherichia coli*, *Pseudomonas aeruginosa*, *Staphylococcus aureus*, *Staphylococcus haemolyticus*, *Klebsiella pneumoniae*, *Staphylococcus epidermidis*. Their dual presence suggests that both habitats function as persistent AMR reservoirs.

From a public health perspective, *S. aureus*, *K. pneumonia* pose zoonotic risks, with potential transmission to farm workers [61, 62]. Additionally, *E. coli* contribute to economic losses due to systemic infections and reduced flock productivity, while also being major foodborne pathogens [3]. Notably, the fact that *P. aeruginosa* and *K. pneumonia* are frequently implicated in hospital-acquired infections highlights their cross-sectoral health impact [63, 64].

The presence of multidrug-resistant organisms such as *P.aeruginosa*,* E.coli*,* S. aureus* and *A. baumannii* in duck farm indoor environments, raises concerns about potential dissemination beyond farm boundaries through aerosols, water runoff, or direct contact with equipment and personnel [65, 66]. These species are also well-documented opportunistic human pathogens, particularly in immunocompromised individuals [57, 67–69] thus their presence in duck farm environments raises concerns about potential zoonotic transmission, especially for farmworkers.

In our dataset, the relative abundance of aaAMRs was lower in treated than in antibiotic-free settings. A plausible, non-exclusive interpretation is that, in these production contexts, prophylactic treatments may reduce the establishment and persistence of high-risk carriers, animals that would otherwise shed resistant bacteria at disproportionately high levels and thereby decrease the net release of resistant biomass into the environment [70, 71]. In other words, although antibiotics impose a transient selective pressure, they may simultaneously suppress overall bacterial burden and shorten the time window during which high-shedding, resistance-enriched carriers dominate, so the environment-facing resistance signal can still decline. Importantly, this pattern should not be read as a universal “antibiotics reduce resistance” rule; rather, it is consistent with a scenario in which the environmental reservoir reflects the balance between selection and total shedding/biomass export, which can shift downward when chronic colonisation and sustained environmental seeding are effectively curtailed under treated conditions.

To strengthen our findings, we also narrowed our focus to differentially abundant aaAMRs with a relative frequency above 0.001 and we identified the AMR carrier species associated with these resistance determinants, allowing for a more detailed examination of potential reservoirs.

A rearing-specific distribution of differentially abundant aaARGs (relative frequency > 0.001) was evident between broiler chickens and ducks. In ducks, 26 aaARGs were identified, of which 23% were overrepresented in antibiotic-free environments and 77% in antibiotic-treated environments. By contrast, in broilers, no differentially abundant aaARGs were detected under antibiotic-treated conditions; all 20 aaARGs identified were significantly enriched in antibiotic-free settings. A lower overall resistome burden under judicious, short courses can co-exist with enrichment of particular loci (aaARGs) in ducks, thus treatment may reduce total ARG signal yet selectively raise some high-fitness determinants in moisture-stabilized biofilms, whereas in broilers (fecal litter-centric reservoir), antibiotic-free cycles likely permit ARG proliferation within the host-associated compartment, which ultimately might seed the environment.

In antibiotic-free broiler houses, *Pseudomonas aeruginosa* was associated with differentially abundant aaARGs in 65% of instances, whereas in antibiotic-treated duck houses the corresponding figure was 50%, underscoring *P. aeruginosa* as a recurrent environmental scaffold for resistance signatures. In antibiotic-free duck environments, *Acinetobacter lwoffii* was detected in 66.7% of cases and *Stenotrophomonas maltophilia* in 20%, indicating a distinct set of environmental opportunists under no-AMU conditions.

Across both broilers and ducks, two-thirds of newly acquired resistances were found in antibiotic-free environments, whereas NAeR counts dropped sharply with targeted treatments. In broilers, Enrocin alone yielded just over 10% NAeRs, and Vertisulf Pulvis + Enrocin < 10%; in ducks, ~ 83% of NAeRs occurred in antibiotic-free settings, with < 10% under Vertisulf Pulvis, < 5% under Floron, and minimal under Avifenicol suggesting that antibiotic-free conditions can foster niches that retain and exchange novel resistance determinants via microbial competition and HGT, whereas disciplined, prophylactic courses constrain additional acquisitions, either by narrowing community turnover or by favoring dominance of a limited set of strains that suppress further ARG accrual.

In broiler antibiotic-free cohorts, *Acinetobacter baumannii*-linked β-lactam resistances (penams, carbapenems, cephalosporins) predominated, and *Bacteroides thetaiotaomicron* contributed streptogramin/macrolide profiles. In ducks, *Bacteroides fragilis* was pivotal for tetracycline resistance under antibiotic-free conditions, while *P. aeruginosa*–associated β-lactam resistance was most pronounced in treated environments (though detectable without treatment). *Clostridium botulinum* emerged as a multi-class NAeR carrier (lincosamide, streptogramin, oxazolidinone, phenicol) under florfenicol exposure, with persistence even in no-AMU settings, highlighting a biofilm-stabilized, multi-resistant phenotype of biosecurity relevance.

Notably, our prior longitudinal metagenomic study [42] on which the present analysis builds showed concordant fecal litter-centric signals: in antibiotic-free broiler flocks, fecal litter harbored >⅔ of emergent resistances dominated by *(A) baumannii* β-lactams (with *(B) thetaiotaomicron* driving streptogramin/macrolide); in ducks, *B. fragilis*–associated tetracycline resistance characterized antibiotic-free fecal litter, while *P. aeruginosa* β-lactam resistance appeared chiefly under treated cycles.

## Conclusion

Within a source-sink framework, reservoir dominance proved system-specific; ducks are environment-centric, while broilers are fecal litter-centric underscoring that control measures must be matrix-targeted, preferring waterline/biofilm management in ducks, versus litter/manure and dust control in broilers.

Although the total number of ARG types was nearly identical between the systems, ducks exhibited marked compartmental divergence, with substantially more environment-unique ARG types and higher environmental richness than broilers.

Above average ARG carrier species overlapped across host and environment in roughly two-thirds of broiler observations but only half of duck observations indicating tighter host-environment exchange in broilers suggesting that duck production requires dual-matrix surveillance.

Shared ARG abundance accumulated progressively across the grow-out cycle, peaking immediately prior to depopulation. The identification of a sizable shared environmental ARG pool, a regional “commons” suggests significant potential for cross-system exchange and dissemination, underscoring that surveillance must extend beyond individual farms. Accordingly, mitigation strategies should target not only on-farm habitats but also transport vehicles, crates, equipment, and broader supply-chain networks where resistant determinants can circulate. Finally, the recurrent detection of high-risk, clinically relevant cross-habitat ARG carriers, including *Pseudomonas aeruginosa*, *Escherichia coli*, *Acinetobacter baumannii*, *Staphylococcus aureus*, *Klebsiella pneumoniae*, *Stenotrophomonas maltophilia*, and toxigenic *Clostridium* spp. underscores the dual threat of zoonotic spillover and food-chain contamination, reinforcing the need for more integrated One Health surveillance and control strategies.

## Materials and methods

### Birds and housing

The parameters described in this section are consistent with those reported in our previously published study [42]. For clarity and reproducibility, we summarize the core methodological details related to animal species, housing conditions, and rearing practices. This study was conducted on Ross 308 broiler chickens and Cherry Valley ducks raised under intensive commercial production conditions at Hungerit Zrt.‘s farms in Kistelek, Felgyő, and Rém, Hungary (GPS coordinates: 46.65513817352346, 20.271366227943012) between 2022 and 2024. To enable direct comparison between the two production systems, fecal litter and environmental (farm indoor surface) samples were collected from parallel housing units: sheds no. 9 and 10 for broilers, and sheds no. 4 and 5 for ducks. One representative cycle per species, broiler cycle 4 (February 14 - March 23, 2023) and duck cycle 2 (September 15 - October 25, 2022), was selected for full longitudinal monitoring. Both broilers and ducks were housed in thermostatically controlled environments and managed in accordance with standardized commercial practices, including defined lighting schedules, phase-specific feeding regimes, routine vaccinations, and strict biosecurity protocols. Broilers were raised in floor pens with wood shavings, while ducks were kept in fully enclosed confinement housing.

Hungerit Zrt. operates a vertically integrated production system with in-house breeding and hatchery operations, ensuring that day-old chicks and ducklings are reared under uniform and traceable conditions. To further standardize comparisons, both species were managed through three defined rearing phases, starter, grower, and finisher, each lasting two weeks. Ducks were fed ‘Duck-pre-starter’ and ‘Duck-starter’ feeds during the first and second weeks, respectively, followed by ‘Duck-grower I’ and ‘Duck-grower II’ feeds. Broilers received ‘Chick-starter’ during the starter phase, ‘Chick-grower I’ and ‘Chick-grower II’ during the grower phase, and ‘Chick-finisher’ during the final two weeks. Biological and environmental samples were collected in parallel using disposable textile overshoes (boot swabs). For manure sampling, boot swabs were worn while systematically walking through animal pens to collect surface-associated microbiota from areas where birds were actively present and defecating. Environmental samples were collected by traversing adjacent service corridors, areas routinely accessed by personnel but not by animals, in order to capture microbial signatures from surfaces representing indirect exposure. Sampling was conducted daily, and subsamples were pooled weekly, with each composite sample representing seven consecutive days (Monday through Sunday). Samples were stored at -80 °C until processing.

All procedures were approved by the institutional ethics committee of the University of Debrecen (permit no. 5/2021/DEMÁB) and carried out in full compliance with applicable national and international animal welfare regulations.

### Antibiotic treatment

The antibiotic treatment protocols described in this section are consistent with those detailed in our previously published study [42]. For clarity and conciseness, we provide a summary of the key aspects. Antibiotic use in broiler chickens and ducks during the study adhered to EU regulations and veterinary instructions, with treatments conducted in parallel sheds using the maximum recommended therapeutic doses. In broiler chickens, antibiotic treatments were applied during the 1st, 2nd, and 8th stocking periods, accounting for an average of 11.90% ± 2.38% of each stocking period. During the 1st stocking period, broilers received 10.99 mg of Sulfacloroquine-Na and Trimethoprim (Vertisulf pulvis) per animal per day for three days. The average body weight ranged from 0.18 to 0.5 kg, with 25,392 birds treated. In addition, during the second treatment, an average antibiotic dosage of 10.01 mg Enrofloxacin per animal per day (Enrocin) was administered for three days. The average body weight ranged from 0.9 to 1.2 kg, and the average number of avians was 24,986. In the 2nd stocking period, an average dosage of 5.52 mg of Enrofloxacin per animal per day was administered for four days, with birds weighing between 0.46 and 0.64 kg and a total of 25,382 birds treated. During the 8th stocking period, 1.65 mg of Enrofloxacin per animal per day was administered for five days to 31,500 birds, with body weights ranging from 0.1 to 0.21 kg.

In ducks, antibiotic treatments occurred during the 2nd, 5th, and 7th stocking periods, representing an average of 10.32% ± 2.75% of each stocking period. During the 2nd stocking period, ducks received 14.47 mg of Florfenicol (Avifenicol) per animal per day for five days, with body weights ranging from 0.7 to 1.5 kg, and 5,668 birds treated. In the 5th stocking period, 17.44 mg of Florfenicol (Floron) per animal per day was administered for five days to 6,888 birds, with body weights between 0.74 and 1.42 kg. During the 7th stocking period, 48.17 mg of Sulfacloroquine-Na and Trimethoprim per animal per day was administered for three days, with body weights from 1.4 to 2.1 kg, treating a total of 7,473 ducks.

For antibiotic-treated animals, the mandatory withdrawal period was observed, after which the animals were also sent to slaughter.

### Sample collection

Biological and environmental samples were collected weekly throughout all rearing phases, starter, grower, and finisher. Sampling was conducted throughout all eight broiler and seven duck stocking periods. However, continuous monitoring was specifically carried out during broiler cycle 4 and duck cycle 2. In the remaining cycles, although sampling occurred regularly, analysis focused on samples from the final week (finisher), representing pre-slaughter conditions.

Each weekly pool comprised seven daily subsamples (Monday to Sunday). In total, 96 weekly composite samples were generated: 50 from broiler cycles (26 biological and 24 environmental samples), 46 from duck cycles (24 biological and 22 environmental samples).

While six weekly pools (weeks 1–6) were collected per cycle, only the final-week samples were sequenced in 13 of 15 cycles. Exceptionally, all six weekly samples were sequenced for broiler cycle 4 and duck cycle 2, providing high-resolution longitudinal insight into microbiome dynamics across all rearing phases.

### Sample preparation and mechanical cell lysis

The sample preparation and mechanical cell lysis protocols presented in this section align with those reported in our previously published study [42]. Below, we provide a concise summary of the key steps. Bacterial cell suspensions were prepared from pooled biological (manure) and environmental (indoor surfaces) shoe cover samples, with each pool comprising seven subsamples. Samples were homogenized in sterile PBS (Biosera, France) for 30 min at 240 × g and 30 °C using an Innova 40 shaker. The supernatants, obtained after centrifugation at 10,000 × g for 10 min (Eppendorf 5810 R), were discarded, and bacterial pellets were resuspended in sterile PBS. Aliquots (1 mL) of the suspensions were transferred to PowerBead tubes (Qiagen, Germany) for mechanical lysis. Prior to lysis, samples were centrifuged twice at 16,000 × g for 5 min (Eppendorf 5418), discarding the supernatants between cycles. The pellets were resuspended in 1000 µL of InhibitEX buffer (Qiagen) and disrupted using a MagNA Lyser (Roche) at 4,000 × g for 30 s, followed by incubation at 4 °C for 2 min, and a second lysis cycle. Heat lysis was performed at 95 °C and 800 × g for 7 min (Eppendorf ThermoMixer^®^ C). Finally, the lysates were centrifuged at 15,000 × g for 1 min, and 400 µL of supernatant was carefully transferred to new microcentrifuge tubes without disturbing the pellet.

### DNA extraction

DNA extraction was carried out like previously described by our group [42]. Shortly, DNA extraction from bacterial cell suspension samples was carried out using the QIAamp Fast DNA Stool Mini Kit (Qiagen, Germany, Cat. 51604) with minor protocol modifications. Briefly, samples were treated with Proteinase K and Buffer AL, vortexed, and incubated at 70 °C for 10 min. Following ethanol addition, lysates were loaded onto QIAamp Mini Spin Columns, washed sequentially with Buffers AW1 and AW2, and dried by centrifugation. DNA was eluted in 50 µL Buffer AE and stored at -20 °C. Concentrations were determined fluorometrically with a Qubit^®^ HS dsDNA Assay Kit (Thermo Fisher Scientific, USA) on a Qubit^®^ 4.0 fluorometer.

### Sequencing and metagenomic data processing

The sequencing and metagenomic data processing methods align with those presented in our previously published study [42], with a summary provided below. Shotgun metagenomic sequencing was performed on an Illumina NovaSeq 6000 instrument (Illumina, USA) with a 150-bp paired-end run at Novogene Bioinformatics Technology (Beijing, China), generating a minimum of 20 million reads per sample. To achieve the required read depth, samples were re-isolated until reaching the neccessary purity (OD260/280 = 1.8-2.0) and concentration (≥ 10 ng/µL). Microbial bioinformatics analysis was performed using the SqueezeMeta pipeline (v1.6.3) with the co-assembly option and no binning [72, 73]. Raw reads underwent quality filtering via Trimmomatic (v0.39) with the parameters: LEADING:8, TRAILING:8, SLIDINGWINDOW:10:15, and MINLEN:30 [74]. Paired-end reads were assembled with MEGAHIT, and taxonomic classification was conducted using DIAMOND (v2.19) against the GenBank nr database with a fast LCA algorithm [75, 76]. For taxonomic assignment, only hits with an e-value ≤ 1e-3 were considered. Identity cutoffs were applied for different taxonomic ranks, requiring a minimum of 85%, 60%, 55%, 50%, 46%, 42%, and 40% identity for species, genus, family, order, class, phylum, and super kingdom, respectively. The analyses were executed on the KIFÜ Hungarian High-Performance Computing Competence Center (HPC CC) Komondor HPC cluster, utilizing 48 CPU cores and 90 GB of RAM per sample. Antibiotic resistance profiling was performed with KneadData for quality control (using Trimmomatic and Bowtie2), and the resistome was predicted using RGI software with the CARD database [77–80]. The list of resistance gene names, ARO names and the related pathogens are included in Supplementary File 3.

### Statistical analysis and data visualization

Continuous variables were reported as the mean ± standard deviation. The Wilcoxon rank-sum test was applied to statistically compare continuous variables. No corrections for multiple comparisons were applied in order to preserve potentially valuable findings and enable independent interpretation from various perspectives, while acknowledging that this methodological choice carries limitations in terms of statistical inference. All statistical tests were two-tailed, with a significance level set at *P* < 0.05. Species richness and evenness of the samples were assessed by calculating the Shannon index, based on the species profile, using the ‘phyloseq’ v.1.44 package in R software [81, 82]. The graphs were generated using the ‘ggplot2’ R package (version 3.5.0) [83, 84], and Venn diagrams were generated with ‘limma’ R package (version 3.28.14) [85]. Radar plots were generated using ‘fmsb’ package [86]. For the Sankey diagram ‘plotly’ package was used [87].

## Supplementary Information

Below is the link to the electronic supplementary material.

Supplementary Material 1

Supplementary Material 2

Supplementary Material 3

## Data Availability

All sequence data used in the analyses were deposited in the Sequence Read Archive (SRA) (http://www.ncbi.nlm.nih.gov/sra) under PRJNA1194338.
