# Supplementary material for: Longitudinal source-sink dynamics of fecal litter and farm indoor environmental resistomes in broiler chicken and Cherry Valley ducks
Source: Anim Microbiome. 2026 Mar 9;8:45. doi: 10.1186/s42523-026-00544-x (PMC13085657; doi:10.1186/s42523-026-00544-x)
Supplement: Supplementary file 2 — Supplementary Material 2 [file 42523_2026_544_MOESM2_ESM.docx]

| **Nr.** | **Category** | **Species** | **Biofilm formation** | **Status of biofilm formation** | **Reference** |
| --- | --- | --- | --- | --- | --- |
| 1 | DUCK and CHICKEN | *Pseudomonas aeruginosa* | YES | Strong | <https://www.mdpi.com/2076-2615/12/4/422> (animal)  <https://pmc.ncbi.nlm.nih.gov/articles/PMC8950561/> (strong biofilm formation) |
| 2 | DUCK | *Vibrio cholerae* | YES | Strong | <https://www.microbiologyresearch.org/content/journal/micro/10.1099/mic.0.040196-0> (human)  <https://pmc.ncbi.nlm.nih.gov/articles/PMC10726288/> (animals)  <https://doi.org/10.3389/fcimb.2018.00120> (strong biofilm formation) |
| 3 | DUCK and CHICKEN | *Corynebacterium striatum* | YES | Strong | <https://pmc.ncbi.nlm.nih.gov/articles/PMC8216692/> (human)  <https://link.springer.com/article/10.1007/s10482-019-01265-9> (human)  <https://pmc.ncbi.nlm.nih.gov/articles/PMC4489456/> (human)  <https://doi.org/10.1186/s12941-022-00546-y> (strong biofilm formation) |
| 4 | DUCK | *Acinetobacter baumannii* | YES | Strong | <https://academic.oup.com/femsre/article/37/2/130/622880?login=true> (human & animal)  <https://doi.org/10.1073/pnas.1800961115> (strong biofilm formation)  <https://doi.org/10.1016/j.micpath.2021.104922> (strong biofilm formation) |
| 5 | DUCK | *Enterococcus faecalis* | YES | Strong | <https://www.frontiersin.org/journals/cellular-and-infection-microbiology/articles/10.3389/fcimb.2022.868338/full>  <https://pubmed.ncbi.nlm.nih.gov/22445820/> (human & animal)  <https://doi.org/10.1099/jmm.0.000647> (strong biofilm formation) |
| 6 | DUCK and CHICKEN | *Erysipelothrix rhusiopathiae* | YES | Medium | <https://journals.asm.org/doi/10.1128/jb.185.9.2739-2748.2003> |
| 7 | DUCK | *Acinetobacter sp* | YES | ------- | <https://www.sciencedirect.com/science/article/pii/S1878818119319310> |
| 8 | DUCK and CHICKEN | *Mammaliicoccus lentus* | YES | Moderate | <https://doi.org/10.5101/nbe.v10i2.p97-103> (human)  <https://pmc.ncbi.nlm.nih.gov/articles/PMC11563631/> (moderate biofilm formation) |
| 9 | DUCK and CHICKEN | *Corynebacterium glutamicum* | YES | Moderate | <https://doi.org/10.3389/fmicb.2022.983545> |
| 10 | DUCK and CHICKEN | *Bifidobacterium adolescentis* | YES | Moderate | <https://www.sciencedirect.com/science/article/pii/S0378111922002682?via%3Dihub>  <https://doi.org/10.1016/j.lwt.2021.111205> (moderate biofilm formation) |
| 11 | DUCK | *Salmonella enterica* | YES | Moderate | <https://doi.org/10.3389/fcimb.2020.624622>  <https://doi.org/10.1016/j.ram.2021.10.003>  <https://doi.org/10.3390/microorganisms9010043> (moderate biofilm formation) |
| 12 | DUCK and CHICKEN | *Staphylococcus aureus* | YES | Strong | <https://doi.org/10.3390/antibiotics11060772>  <https://doi.org/10.3389/fcimb.2023.1137947> (strong biofilm formation) |
| 13 | DUCK and CHICKEN | *Vibrio fluvialis* | YES | Strong | <https://pmc.ncbi.nlm.nih.gov/articles/PMC3636005/>  <https://doi.org/10.3389/fmicb.2014.00091> (strong biofilm formation) |
| 14 | DUCK and CHICKEN | *Nocardia farcinica* | NO |  | ----------------------------------------------- |
| 15 | DUCK and CHICKEN | *Klebsiella pneumoniae* | YES | Strong | <https://doi.org/10.3389/fmicb.2023.1238482>  <https://doi.org/10.3389/fcimb.2023.1137947> (strong biofilm formation) |
| 16 | CHICKEN | *Escherichia coli* | YES | Moderate | <https://doi.org/10.3389/fmicb.2022.841516> (animal)  <https://doi.org/10.1128/AEM.01126-21> (animal)  <https://doi.org/10.3390/microorganisms11122858> (moderate biofilm foramtion) |
| 17 | CHICKEN | *Staphylococcus haemolyticus* | YES | Strong | <https://doi.org/10.1128/JCM.01891-08> (human)  <https://doi.org/10.1128/JCM.01891-08> (strong biofilm formation) |
| 18 | CHICKEN | *Butyrivibrio fibrisolvens* | NO |  | --------------------------------------- |
| 19 | CHICKEN | *Enterococcus faecium* | YES | Strong | <https://doi.org/10.1186/s12866-023-02834-9> (environment)  <https://doi.org/10.3390/pathogens12091101> (strong biofilm formation) |
| 20 | CHICKEN | *Bifidobacterium bifidum* | YES | Moderate | <https://doi.org/10.3390/ijms22147596>  <https://doi.org/10.1016/j.lwt.2021.111205> (moderate biofilm formation) |
| 21 | CHICKEN | *Staphylococcus epidermidis* | YES | Strong | <https://pmc.ncbi.nlm.nih.gov/articles/PMC2903046/>  <https://www.sciencedirect.com/science/article/pii/S0921448818305789> (animal)  <https://doi.org/10.3389/fcimb.2023.1137947> (strong biofilm formation) |
| 22 | CHICKEN | *Brachybacterium paraconglomeratum* | NO |  | ------------------------------------------- |
| 23 | CHICKEN | *Stenotrophomonas maltophilia* | YES | Strong | <https://doi.org/10.3390/microorganisms9010049>  <https://doi.org/10.1016/j.genrep.2020.100827> (strong biofilm formation) |
| 24 | CHICKEN | *Megasphaera elsdenii* | NO |  | --------------------------------------------- |
| 25 | CHICKEN | *Geobacillus stearothermophilus* | YES | Strong | <https://doi.org/10.1016/B978-0-12-384730-0.00020-3> (environment)  <https://pmc.ncbi.nlm.nih.gov/articles/PMC8091125/> (environment)  <https://doi.org/10.1016/j.ijfoodmicro.2021.109318> (strong biofilm formation) |

| Strong biofilm formation | Moderate biofilm formation |
| --- | --- |
| *Pseudomonas aeruginosa* | *Erysipelothrix rhusiopathiae* |
| *Vibrio cholerae* | *Mammaliicoccus lentus* |
| *Corynebacterium striatum* | *Corynebacterium glutamicum* |
| *Acinetobacter baumannii* | *Bifidobacterium adolescentis* |
| *Enterococcus faecalis* | *Salmonella enterica* |
| *Staphylococcus aureus* | *Escherichia coli* |
| *Vibrio fluvialis* | *Bifidobacterium bifidum* |
| *Klebsiella pneumoniae* |  |
| *Staphylococcus haemolyticus* |  |
| *Enterococcus faecium* |  |
| *Staphylococcus epidermidis* |  |
| *Stenotrophomonas maltophilia* |  |
| *Geobacillus stearothermophilus* |  |

**Supplementary File 2. Tables of biofilm-forming taxa, classified into moderate and high biofilm-forming capacity**
